# Effect of compressed TiO_2_ nanoparticle thin film thickness on the performance of dye-sensitized solar cells

**DOI:** 10.1186/1556-276X-8-459

**Published:** 2013-11-05

**Authors:** Jenn Kai Tsai, Wen Dung Hsu, Tian Chiuan Wu, Teen Hang Meen, Wen Jie Chong

**Affiliations:** 1 Department of Electronic Engineering, National Formosa University, Yunlin 632, Taiwan; 2 Department of Materials Science and Engineering, National Cheng Kung University, Tainan City 701, Taiwan

**Keywords:** Mechanism compression, Thickness, Dye-sensitized solar cells (DSSCs), TiO_2_, Doctor blading method

## Abstract

In this study, dye-sensitized solar cells (DSSCs) were fabricated using nanocrystalline titanium dioxide (TiO_2_) nanoparticles as photoanode. Photoanode thin films were prepared by doctor blading method with 420 kg/cm^2^ of mechanical compression process and heat treatment in the air at 500°C for 30 min. The optimal thickness of the TiO_2_ NP photoanode is 26.6 μm with an efficiency of 9.01% under AM 1.5G illumination at 100 mW/cm^2^. The efficiency is around two times higher than that of conventional DSSCs with an uncompressed photoanode. The open-circuit voltage of DSSCs decreases as the thickness increases. One DSSC (sample D) has the highest conversion efficiency while it has the maximum short-circuit current density. The results indicate that the short-circuit current density is a compromise between two conflict factors: enlargement of the surface area by increasing photoanode thickness and extension of the electron diffusion length to the electrode as the thickness increases.

## Background

Most solar cells are fabricated using Si-based materials [1]; however, in recent years, new materials have been discovered to replace Si for applications in solar cells. A dye-sensitized solar cell (DSSC) [2-4] is one of the alternatives as it is low cost and lightweight and can be fabricated on flexible substrates to improve portability. DSSC also shows high energy conversion efficiency by using nanoparticle (NP) thin film as photoanode. The film has a nonporous structure, which has an extremely large specific surface area that enhances dye adsorption as well as light harvesting. Titania (TiO_2_) nanoparticle is stable and nontoxic and has relatively high transmittance in the visible spectrum, thus becomes a promising nanoparticle material for applications in DSSCs. The band gap of rutile- and anatase-phase TiO_2_ is 3.0 and 3.2 eV, respectively. The anatase phase is specially preferred due to its good photocatalytic properties and wide direct band gap [5,6].

A dye-sensitized solar cell is composed of three main structures: (1) a dye sensitizer whose function is to harvest solar energy and generate excitons [7,8], (2) a nanostructured metal oxide to transport electrons efficiently [9,10], and (3) a redox electrolyte or hole-transporting material [11,12]. The key element in a DSSC is the photoanode, which is composed of a thin film of TiO_2_ NPs. Though the nanoparticle thin film has a high specific surface area, electron percolation is hindered by limited interconnected NPs resulting in photoelectron loss due to recombination between the photoelectrons and the oxidized dye molecules or electron-accepting species in the electrolyte. To solve this issue, mechanical compression of the photoanode thin film was adopted to increase the effective interconnection between NPs. The optimal thickness of the mechanically compressed TiO_2_ nanoparticle thin film was reported.

## Methods

### Experimental details

#### Deposition of TiO_2_ thin film as photoanode

TiO_2_ paste (10 wt%) was prepared by mixing nanocrystalline TiO_2_ nanoparticles (TG-P25, Degussa, Shinjuku, Tokyo, Japan; the average nanoparticle diameter was about 25 to 30 nm) with *tert*-butyl alcohol and deionized water. The TiO_2_ paste was then scraped on a transparent fluorine-doped tin oxide (FTO) glass of 8-Ω/sq resistivity by doctor blading method. The films were mechanically compressed with a pressure of 420 kg/cm^2^. After the compression, the films were annealed in air by two consecutive steps: 150°C for 90 min and 500°C for 30 min. The 150°C annealing is to decompose residual organic compounds, and the 500°C annealing is to assist the interconnection of TiO_2_ NPs.

#### DSSC fabrication

**Figure 1:**
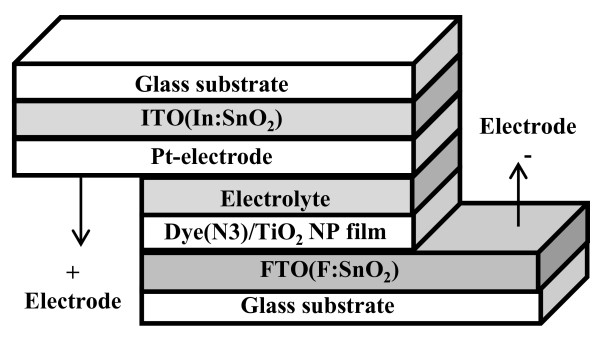
**The schematic structure of the dye-sensitized solar cell with TiO**_**2 **_**nanoparticle thin film as photoanode.**

Figure 1 shows the structure of the dye-sensitized solar cell with TiO_2_ NP thin film as photoanode. The compressed TiO_2_ NP thin films were immersed in 0.3 mM N3 dye (*cis*-bis(dithiocyanato)-bis(4,4*'*-dicarboxylic acid-2,2*'*-bipyridine) ruthenium(II)) for 5 h. Subsequently, they were rinsed in acetonitrile for a few seconds to wash out unbound dyes and then dried in the oven at 45°C. The Pt thin film as counter electrode was grown on an indium tin oxide (ITO) glass by an electroplating process. The FTO substrate with deposited compressed TiO_2_ NP thin film with adsorbed dyes was then bonded to the ITO glass with Pt counter electrode using a 50-μm-thick hot-melt polymer spacer. Sealing was accomplished by pressing the two electrodes together at about 115°C for a few seconds. The redox electrolyte, consisting of 0.5 M LiI, 0.05 M I_2_, 0.5 M 4-*tert*-butylpyridine (TBP), and 1 M 1-propy1-2,3-dimethylimidazolium (DMPII) mixed into 3-methoxypropionitrile (MPN), was injected into the cell by capillary forces through an injecting hole, previously made in the counter electrode using a drilling machine. Finally, the hole was covered and sealed with a piece of hot-melt polymer, preventing the leakage of the fluid-type electrolyte. The resulting active electrode area was approximately 0.25 cm^2^ (0.5 cm × 0.5 cm).

#### Characterizations and photoelectrochemical measurement

The structures and morphologies of the TiO_2_ NP thin films were studied using a field emission scanning electron microscope (FESEM; JSM-7500F, JEOL, Akishima-shi, Japan). The ultraviolet–visible (UV–vis) transmittance spectrum of the sample was observed using a UV–vis spectrophotometer (U-2900, Hitachi High-Technologies Corporation, Tokyo, Japan). Electrochemical impedance spectroscopy (EIS; Zahner Zennium, Kronach, Germany), which is a standard method to measure the current response under an ac voltage of various frequencies, was used to characterize the carrier transport behavior of the DSSCs. The frequencies ranged from 10 mHz to 100 kHz. The measurement was under illumination of air mass 1.5 global (AM 1.5G) at an applied bias of open-circuit voltage. The incident photon-to-current conversion efficiency (IPCE), which was determined by the light-harvesting efficiency of the dye, the quantum yield of electron injection, and the efficiency of collecting the injected electrons, was recorded using an IPCE instrument equipped with a 1,000-W xenon arc lamp as the light source composed of a compact 1/8-m monochromator (CM110, Spectral Products, Putnam, CT, USA), a color filter wheel (CFW-1-8, Finger Lakes Instrumentation, Lima, NY, USA), and a calibrated photodiode (FDS1010-CAL, Thorlabs Inc., Newton, NJ, USA). The IPCE data were taken using a source meter (2400, Keithley Instruments, Inc., Cleveland, OH, USA) with lluminating monochromatic light on the solar cells (with the wavelength from 300 to 800 nm). The current–voltage characteristics of the samples were measured using the Keithley 2400 source meter under a simulated sunlight (SAN-EI XES-40S1, San Ei Brand, Higashi-Yodogawa, Japan), with AM 1.5G radiation at 100 mW/cm^2^.

## Results and discussion

**Figure 2:**
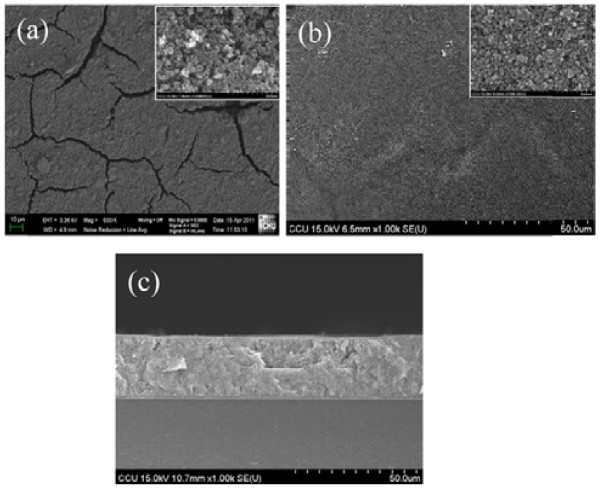
**FESEM images of TiO**_**2 **_**nanoparticle thin film on FTO glass fabricated by doctor blading method. (a, b)** The top-view images of the as-deposited and the compressed film, respectively. **(c)** The cross-sectional image. The insets in **(a)** and **(b)** are high-magnification images.

Photoanodes of the compressed TiO_2_ NP thin film with various thicknesses were prepared in this study. Samples A to F represent the thickness of the film with 12.7, 14.2, 25.0, 26.6, 35.3, and 55.2 μm, respectively. The thickness is determined by the cross-sectional FESEM images. Figure 2 shows the surface morphology of TiO_2_ NP thin films. The cracks were found in the as-deposited TiO_2_ NP thin film (Figure 2a). The film also showed a porous structure as indicated by the inset of Figure 2a. Several mechanisms have been proposed to explain the crack formation in the as-deposited film, including an influence of capillary forces in a rapid evaporation of solvents from the film surface during the drying process, a decrease of bonding strength among TiO_2_ NPs when the film is very thick, and a mismatch of the thermal expansion between the FTO substrate and the TiO_2_ NP thin film [13-16]. The cracks can be annihilated by subsequent annealing after a mechanical compression of 420 kg/cm^2^ of pressure, as shown in Figure 2b. Comparing Figure 2a, b, the compressed film is homogeneous and smooth which may enhance the electron transport between NPs. Although the compressed film is smooth, there is still a porous structure, as shown in the inset of Figure 2b, which enhances the following dye absorption. The cross-sectional FESEM image of the TiO_2_ NP thin film prepared by doctor blading method with the compression process is shown in Figure 2c. The result indicates that the compressed film is also condensing in the plane-normal direction.

**Figure 3:**
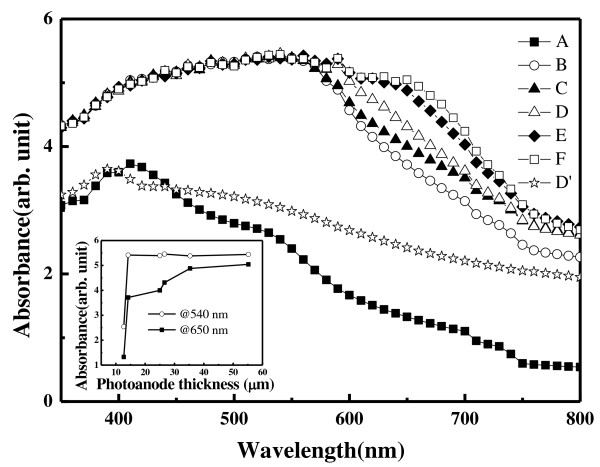
**The UV–vis absorption spectra of compressed TiO**_**2 **_**NP thin films with various thicknesses.** Samples A to F have a photoanode thickness of 12.7, 14.2, 25.0, 26.6, 35.3, and 55.2 μm, respectively, with dye adsorption. Sample D' is the TiO_2_ NP thin film of 26.6 μm in thickness (the same as sample D) but without dye adsorption.

In order to reveal the effect of dyes adsorbed on the TiO_2_ NPs, a compressed TiO_2_ NP thin film with a thickness that is the same as that of sample D (26.6 μm) but without dye adsorption was prepared. Its UV–vis adsorption spectrum was compared with those of samples A to F, as shown in Figure 3. The range of spectral absorbance was between 0 and 6 which is related to air, to which 0 absorbance was assigned. The absorbance of the films with dye adsorption (samples A to F) is larger than that of the films without dye adsorption. The absorbance increases as the thickness increases which may be attributed to the increase of the number of absorbed dye molecules in the TiO_2_ NP thin film. In the short light wavelength region (less than 590 nm), the absorbance is almost the same among samples B to F whose thickness is greater than or equal to 14.2 nm, as shown in the inset of Figure 3. It is because the adsorption characteristic of N3 dye is located at the light wavelength of 540 nm. On the other hand, in the long light wavelength region, the absorbance increases as the thickness increases. The result is shown in the inset of Figure 3 by comparison of the absorbance of samples B to F at 650 nm. It is because long-wavelength light has high transmittance resulting in high absorbance for the thick film.

**Figure 4:**
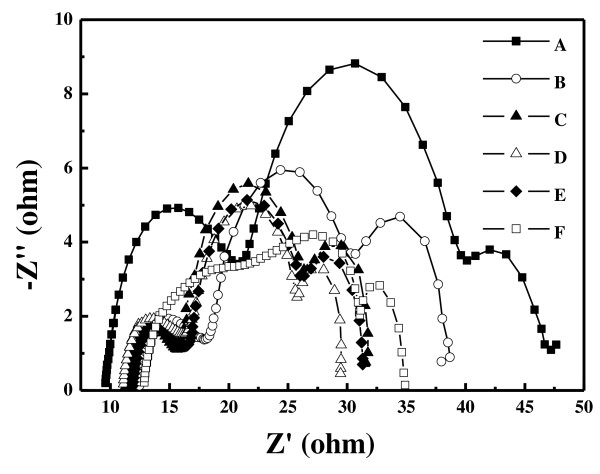
**Nyquist plots of the DSSCs composed of the compressed TiO**_**2 **_**NP thin film as photoanode.** Samples A to F have a photoanode thickness of 12.7, 14.2, 25.0, 26.6, 35.3, and 55.2 μm, respectively, with dye adsorption.

To further understand the electron transport processes in the DSSCs made of TiO_2_ photoanodes, the EIS spectrum was analyzed. Figure 4 shows the Nyquist plots, minus the imaginary part of the impedance -*Z''* as a function of the real part of the impedance *Z'* while the frequency sweeps from 10 mHz to 100 kHz, of samples A to F. Three distinct semicircles are observed, which are attributed to the electrochemical reaction at the Pt counter electrode/electrolyte interface, the charge transport through the TiO_2_/dye/electrolyte interfaces, and the Warburg diffusion process of I^-^/I_3_^-^ in the electrolyte, from left to right, respectively [3,17]. The diameter (*R*_K_) of the middle semicircle corresponds to the resistance associated with the transport of electrons through the dye/TiO_2_ NP photoanode/electrolyte interfaces The *R*_K_ values for samples A to F are listed in Table 1. The result indicates that sample D has the smallest *R*_K_ among the six samples.

**Table 1:** **Characteristics of DSSCs composed of the compressed TiO**_**2 **_**NP thin film as photoanode**

**Sample**	**Thickness**	***R***_**K**_	***V***_**OC**_	***J***_**SC**_	**FF**	***η***
	**(μm)**	**(Ω)**	**(V)**	**(mA/cm**^**2**^**)**	**(%)**	**(%)**
A	12.7	19.2	0.71	12.62	60.89	5.43
B	14.2	12.5	0.68	19.88	57.90	7.80
C	25.0	10.6	0.68	21.59	58.33	8.59
D	26.6	9.41	0.68	22.41	59.66	9.01
E	35.3	9.87	0.66	22.32	56.10	8.30
F	55.2	10.1	0.62	19.37	54.67	5.85

**Figure 5:**
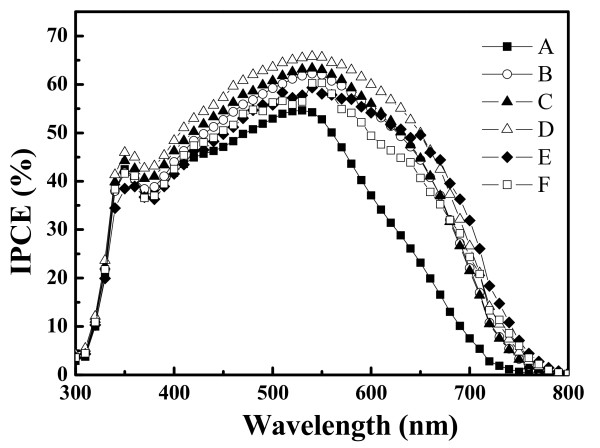
**IPCE characteristics of the DSSCs composed of the compressed TiO**_**2 **_**NP thin film as photoanode.** Samples A to F have a photoanode thickness of 12.7, 14.2, 25.0, 26.6, 35.3, and 55.2 μm, respectively, with dye adsorption.

Figure 5 shows the IPCE as a function of wavelength. High IPCE represents high optical absorption and hence improves the incident photon-to-electron conversion efficiency. The IPCE results indicate that the wavelength of incident light that contributes to photo-to-current conversion mainly ranges from 300 to 800 nm. This is because the N3 dye has the highest quantum efficiency at the wavelength of 540 nm. Thus, for all the samples, the highest IPCE is observed at 540 nm. Sample D has a quantum efficiency of about 67%, which is approximately 12% higher than that of sample A.

**Figure 6:**
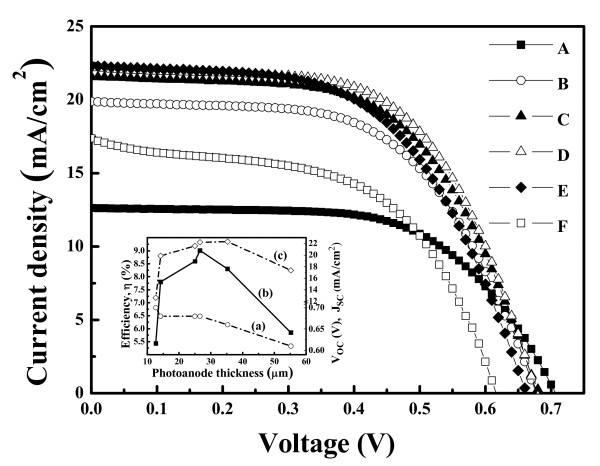
***J*-*V *characteristics of the DSSCs composed of the compressed TiO**_**2 **_**NP thin film as photoanode.** Under AM 1.5G sunlight. The inset shows (a) open-circuit voltage (*V*_OC_), (b) overall photo-to-electron conversion efficiency (*η*), and (c) short-circuit current density (*J*_SC_) as a function of photoanode thickness.

Figure 6 shows the photocurrent density-voltage characteristics of the DCCSs of samples A to F under AM 1.5G. The photovoltaic properties of DSSCs are summarized in Table 1. The open-circuit voltage (*V*_OC_) decreases monotonically as the thickness of TiO_2_ photoanode increases. The result indicates that the recombination rate increases with the increase of photoanode thickness. It is due to the long diffusion distance for the photoelectron to transport to the electrode enhancing the probability of recombination. The short-circuit current density (*J*_SC_), however, does not show simple relations with the thickness, in which sample D has the highest density of 22.41 mA/cm^2^.

In general, the photocurrent density of DSSCs is influenced by three factors: (1) the number of photoexcited electrons, which is dominated by the adsorptive capacity of dye molecules, (2) the recombination rate at the interface of dye/TiO_2_ NP or TiO_2_ NP/electrolyte, and (3) the redox of I^-^/I_3_^-^ in the electrolyte. In this study, the TiO_2_ NP thin film is compressed before heat treatment. The procedure enhances the interconnection between the NPs, hence decreases the recombination probability. The performance of the DSSCs is improved. Besides, a thick photoanode induces a large surface area enhancing dye molecules to adsorb on it. Hence, a thick photoanode captures more light to generate photoexcited electrons. However, the *J*_SC_ requires that these electrons successfully transport to the FTO substrate (electrode) without recombination at the dye/photoanode or photoanode/electrolyte interfaces; therefore, electron diffusion length is also a key point that needs to be considered. Though a thick photoanode enhances the generation of photoexcited electrons, a long electron diffusion length is inevitable for those photoexcited electrons generated in the deep layer. Thus, the *J*_SC_ is a compromise between the two conflict factors: enlarged surface area by increasing photoanode thickness and increased thickness resulting in a long electron diffusion length. The experimental results indicate that the optimized thickness is 26.6 nm. The probability of recombination of injected electrons and the iodides in the electrolyte is smallest in this case. Therefore, sample D has the highest photo-to-electron conversion efficiency of 9.01%. The results also agree with those of EIS and IPCE, as shown in the inset of Figure 6.

## Conclusions

The effect of TiO_2_ NP photoanode thickness on the performance of the DSSC device was studied. The TiO_2_ NP photoanode thin film was fabricated by mechanical compression before thermal treatment. The final film was uniform and dense. The UV–vis spectrophotometer analysis indicates that the absorbance increases with the increase of the thickness of TiO_2_ NP thin film due to the large surface area enhancing the adsorption of dye molecules. However, the optimal incident photon-to-current conversion efficiency and total energy conversion efficiencies were found in the TiO_2_ NP photoanode film with a thickness of 26.6 μm under an incident light intensity of 100 mW/cm^2^. The results indicate that there are two conflict factors acting together so that an optimal thickness is observed. The two factors are as follows: (1) increasing the photoanode thickness could enlarge the surface area and enhance the adsorption of dye molecules which improves the light absorbance as well as the generation of photoexcited electrons and (2) a thick photoanode results in a long electron diffusion distance to the FTO substrate (electrode) which increases the probability of recombination and thus degrades the efficiencies.

## Abbreviations

DSSCs: Dye-sensitized solar cells; EIS: Electrochemical impedance spectroscopy; FESEM: Field emission scanning electron microscopy; FTO: Fluorine-doped tin oxide; IPCE: Incident photon-to-current conversion efficiency; ITO: Indium tin oxide; NPs: Nanoparticles; TiO2: Titanium dioxide; UV–vis: Ultraviolet–visible.

## Competing interests

The authors declare that they have no competing interests.

## Authors’ contributions

JKT designed the work and wrote the manuscript. WJC carried out the preparation of samples, UV–vis absorption, and *I*-*V* measurements. WDH carried out the measurement and analysis of EIS. TCW and THM helped in carrying out the FESEM and IPCE measurements. All authors read and approved the final manuscript.
